# Small Molecule Microcrystal Electron Diffraction for the Pharmaceutical Industry–Lessons Learned From Examining Over Fifty Samples

**DOI:** 10.3389/fmolb.2021.648603

**Published:** 2021-07-12

**Authors:** Jessica F. Bruhn, Giovanna Scapin, Anchi Cheng, Brandon Q. Mercado, David G. Waterman, Thejusvi Ganesh, Sargis Dallakyan, Brandon N. Read, Travis Nieusma, Kyle W. Lucier, Megan L. Mayer, Nicole J. Chiang, Nicole Poweleit, Philip T. McGilvray, Timothy S. Wilson, Michael Mashore, Camille Hennessy, Sean Thomson, Bo Wang, Clinton S. Potter, Bridget Carragher

**Affiliations:** ^1^NanoImaging Services, San Diego, CA, United States; ^2^Department of Chemistry, Yale University, New Haven, CT, United States; ^3^UKRI STFC Rutherford Appleton Laboratory, Didcot, United Kingdom; ^4^CCP4, Research Complex at Harwell, Rutherford Appleton Laboratory, Didcot, United Kingdom; ^5^Biogen, Cambridge, MA, United States

**Keywords:** crystallography, transmission elections microscopy, electron diffraction (ED), MicroED, drug development, structure, small molecule, medicinal chemistry

## Abstract

The emerging field of microcrystal electron diffraction (MicroED) is of great interest to industrial researchers working in the drug discovery and drug development space. The promise of being able to routinely solve high-resolution crystal structures without the need to grow large crystals is very appealing. Despite MicroED’s exciting potential, adoption across the pharmaceutical industry has been slow, primarily owing to a lack of access to specialized equipment and expertise. Here we present our experience building a small molecule MicroED service pipeline for members of the pharmaceutical industry. In the past year, we have examined more than fifty small molecule samples submitted by our clients, the majority of which have yielded data suitable for structure solution. We also detail our experience determining small molecule MicroED structures of pharmaceutical interest and offer some insights into the typical experimental outcomes. This experience has led us to conclude that small molecule MicroED adoption will continue to grow within the pharmaceutical industry where it is able to rapidly provide structures inaccessible by other methods.

## Introduction

The three-dimensional structure of a molecule provides detailed information regarding relative atom positions, bonding and intra-molecular interactions, which conversely informs stability, reactivity, solubility, and ultimately function. For example, in catalytic chemistry, information regarding the catalyst structure and grain morphology greatly helps in mechanism characterization and process optimization (Wennmacher et al., 2020). Knowledge of the active enantiomeric form of pharmaceutical compounds has become essential in recent years, as generally only one enantiomer is active, and the other(s) may be inactive or even unsafe (Ariëns, 1984). Determination of the 3D structure of synthetic organic reaction products or by-products can also allow for the understanding of the reaction mechanism, and possibly optimization of the reaction itself. Structural characterization of small molecules has thus far primarily been carried out using nuclear magnetic resonance (NMR), mass spectrometry (MS), and X-ray diffraction (XRD), with single-crystal XRD being the preferred method when a high degree of confidence in the 3D model is required.

In the development of small molecule pharmaceuticals, knowledge of the crystalline solid form is extremely important, beyond confirmation of the proposed 2D structure. The crystal form adopted by an active pharmaceutical ingredient (API) and the lattice interactions that hold it together can have important ramifications for stability, tableting properties, solubility and dissolution rates, ultimately affecting bioavailability, potency, and even toxicity (Lu and Rohani, 2009; Censi and Di Martino, 2015). It is therefore critically important to understand the solid form(s) of APIs and the crystal lattice interactions that underly them. The majority of APIs are estimated to be able to exist in more than one polymorphic form (Raza et al., 2014). Polymorphs are alternative crystal forms of the same compound with different crystal lattice properties, which can give rise to drastically different drug properties. Selecting the optimal polymorph or other crystal form (salt, solvate, hydrate, or co-crystal) is therefore very important for drug formulation. Additionally, as the vast majority of drug substances currently being developed exhibit solubility issues, formulation chemists are increasingly hard pressed to find crystal forms that enhance solubility. It is therefore highly desirable to be able to easily determine the structure of the many crystal forms an API can adopt in order to understand the underlying lattice properties and better engineer the optimal crystal form for development.

Thus far, the vast majority of small molecule crystal structures have been determined by single-crystal XRD, but a major bottleneck for this method is that it requires the generation of large, well-ordered crystals. For synchrotron radiation sources, the smallest suitable crystals are typically around 5–10 μm, and in-house X-ray sources require crystals about ten times larger than this (Gruene et al., 2018). When suitably sized crystals cannot be produced, X-ray powder diffraction (XRPD) (Thakral et al., 2018) can sometimes be used to determine a three-dimensional structure of the compound. While technical advances over the last 20 years have made it feasible to use XRPD to determine the crystal structures of materials of moderate complexity (Harris, 2012), determining crystal structures from powder diffraction remains significantly more challenging than single crystal diffraction data, particularly in the case of larger organic molecules for which there can be many overlapping peaks in the powder diffraction pattern.

Very recently, microcrystal electron diffraction (MicroED) or 3D electron diffraction using the continuous rotation method has emerged as an attractive alternative technique for structure determination of proteins, peptides, inorganic compounds, and small organic molecules (Shi et al., 2013; Clabbers et al., 2018; Gruene et al., 2018; Jones et al., 2018). Electrons interact very strongly with matter, allowing for data collection from sub-micron sized crystals. This dramatically expands the possibilities for determining structures of crystal forms which do not readily grow large crystals, such as the several thousand APIs for which only crystalline powders are available (Gruene et al., 2018). In many cases, screening for crystallization conditions can be eliminated entirely when MicroED is employed as many compounds readily form crystals during purification, thus saving time and material.

**FIGURE 1:**
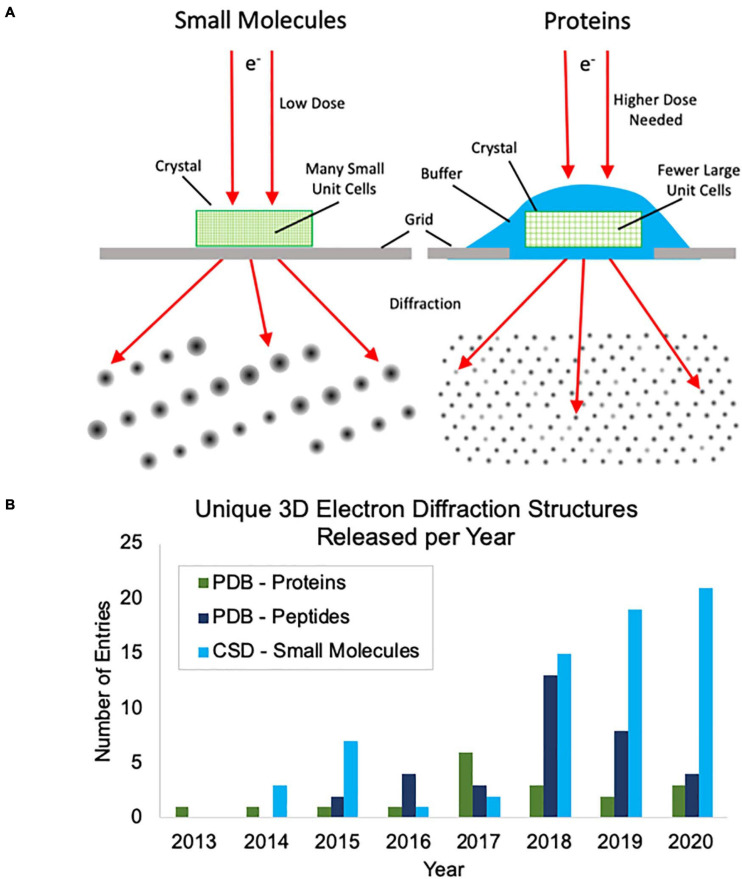
**(A)** Cartoon depiction of the differences between small molecule and protein microcrystal electron diffraction (MicroED). First, protein crystals are grown in aqueous conditions and must be kept hydrated during grid preparation, whereas small molecule crystals are typically dry and can be directly applied to the grid without the need for sophisticated grid vitrification protocols. Second, small molecule crystals are composed of many small unit cells while protein crystals contain fewer, larger unit cells per crystal volume. Because of this, higher doses are typically needed for protein samples to generate diffraction spots of similar intensities. **(B)** The growth of unique structure depositions by year in the Protein Data Bank (PDB) and Cambridge Structural Database (CSD) determined by MicroED or related 3D electron diffraction techniques. Depositions into the PDB are broken up into proteins and peptides. Proteins were defined as having more than 50 amino acids.

Thus far, most of the academic interest in, and development of, MicroED has centered around protein samples where there is a great need for methods to address samples that cannot produce large crystals. Protein MicroED presents unique challenges primarily in the realm of crystal screening, sample grid preparation and data collection. Hydrated crystals are very sensitive to changes in their environment and are generally applied to grids along with the crystallization solution or other buffer. This buffer must then be removed or sufficiently thinned to allow for penetration by the electron beam, without damaging the crystals, and this can be challenging to achieve. Many groups have resorted to using focused ion beams to mill through buffer coated crystals, adding yet another very expensive and labor-intensive step to the process (Duyvesteyn et al., 2018; Martynowycz et al., 2019; Beale et al., 2020). Small molecule crystals fortunately do not pose the same challenges for grid preparation, because they are generally dry powders that can easily be broken into suitably small crystals and can be directly applied to grids (Figure 1A). Additionally, protein crystals generally produce lower intensity diffractions spots at the same electron dose relative to small molecule crystals (Figure 1A) and this results in more radiation damage as the doses that must be used for protein crystal diffraction are generally slightly higher than for small molecules. Taken together, these factors make small molecule MicroED much easier to practically implement than protein MicroED and have likely contributed to the faster growth of small molecule structures being solved by MicroED and other, related 3D electron diffraction techniques. This trend can be seen when comparing the unique protein structures determined by MicroED/3D electron diffraction deposited in the Protein Data Bank (PDB) with the unique peptide structures deposited in the PDB and small molecule structures deposited in the Cambridge Structural Database (CSD) in recent years (Figure 1B).

Despite the rapid growth of the field, small molecule MicroED adoption has been slow, especially in industrial settings. Although many research groups recognize the great potential of small molecule MicroED, widescale adoption is inhibited by steep requirements for instrumentation, infrastructure, and expertise. This is especially the case if only a limited number of projects are contemplated by a single group, making it hard to justify the required capital investments. Improvements in access to service facilities, in speed and automation of data collection and processing, and in the ease with which structure solution and refinement can be completed, will help to make small molecule MicroED far more broadly accessible, especially for researchers in the pharmaceutical industry.

**FIGURE 2:**
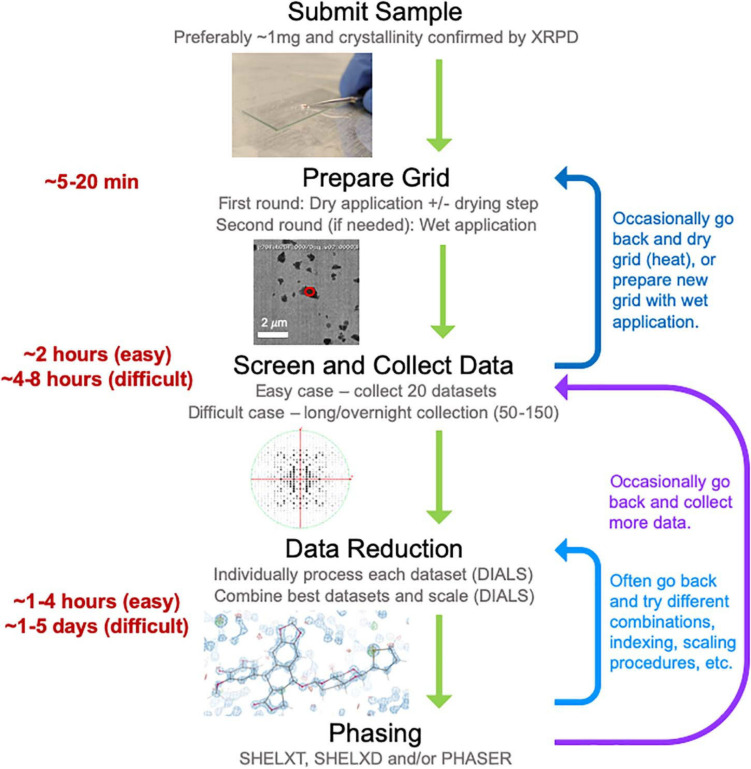
The general workflow pipeline at NIS with typical times given for easy and difficult cases. At the screening and data collection stage, an “easy” target has many crystals that all diffract to high resolution (≤1.1 Å), while a “difficult” target may be much more heterogeneous in terms of diffraction quality and/or may have been flagged as “difficult” if processing the first couple of datasets revealed problematic features (low resolution, radiation damages and/or low completeness upon merging datasets). Data processing, encompassing both data reduction and phasing, is a very iterative process where many data reduction strategies are typically employed before successful phasing can be achieved.

Here we describe our experience establishing a MicroED data collection and structure determination service pipeline (Figure 2) at NanoImaging Services (NIS), a commercial service provider to the biopharmaceutical and biotechnology industries. To establish this pipeline, we adapted our existing data collection software system, Leginon (Suloway et al., 2005), enabling the collection of continuous-rotation, electron-diffraction data with the camera in rolling-shutter mode, incorporating rapid crystal preview evaluation, and fully automating data collection of a queue of pre-selected crystal targets (Cheng et al., 2021). We also established a robust data processing pipeline built on the DIALS (Clabbers et al., 2018) and SHELX (Schneider and Sheldrick, 2002; Sheldrick, 2015b) suites. Using this pipeline, we determined the structures of three small molecule validation targets (progesterone, biotin, and paracetamol) and one novel, proof of concept structure (teniposide), all of which showed excellent refinement statistics relative to other structures determined by electron diffraction methods. Additionally, we have now examined over fifty small molecule samples submitted by our clients. We have also assisted in structure solution for a subset of these samples as requested by our clients. For the vast majority of these pharmaceutical samples, growing crystals large enough for single-crystal XRD was not possible despite the efforts of experienced small molecule crystallographers, and other structural techniques, such as NMR and MS, provided inconclusive results. MicroED is exceptionally well suited to provide structures for important and interesting compounds when other structural techniques are too challenging or provide incomplete information. Based on our experience providing MicroED services to the pharmaceutical industry over the past year, we are confident that MicroED is poised to join XRD, NMR, and MS as a routine tool for small molecule structure determination, especially when targets pose challenges for the traditional, established methods.

## Materials and Methods

### Sample Preparation

**FIGURE 3:**
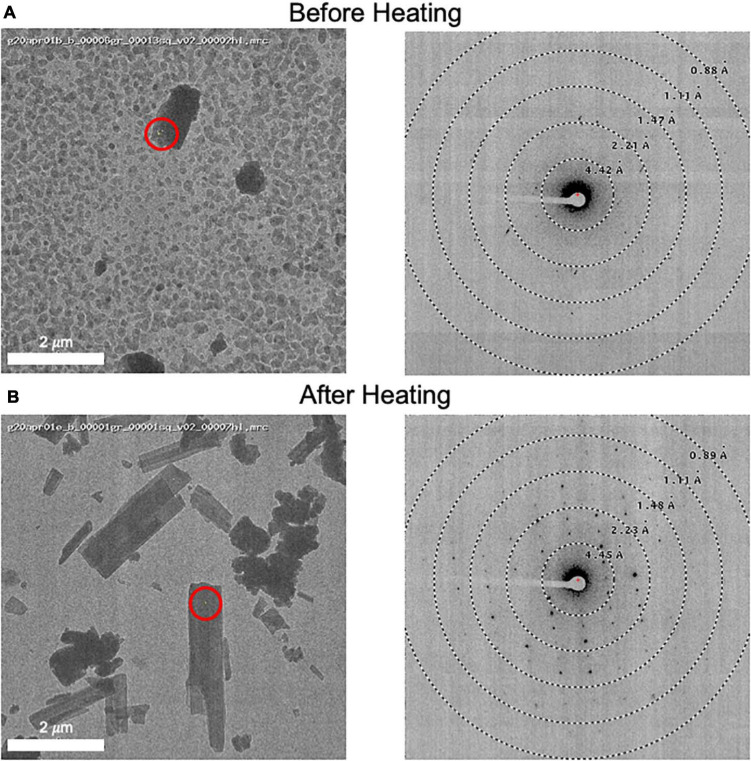
**(A)** An image of a client sample (left) and its diffraction pattern (right). The area targeted is indicated by a red circle showing the approximate beam diameter. Note the grainy background material and poor diffraction. **(B)** This grid was removed from the microscope and subsequently heated to 50°C for 10 minutes prior to re-freezing in liquid nitrogen. An image (left) and its diffraction pattern (right) are shown. Note the disappearance of the grainy background material and the improved diffraction pattern. We also note that the MicroED structure determined from these crystals matches the experimentally collected XRPD pattern and therefore we do not believe that heating caused a form change in this case.

Grids for MicroED data acquisition were prepared as follows: (i), a small amount of dry sample was first ground between two glass slides; (ii) a pure carbon grid (Ted Pella 01840) that had not been plasma-cleaned or glow-discharged was dragged across the powder; (iii) the grid was gently tapped to remove excess powder; and (iv) the grid was clipped at room temperature and transferred to the transmission electron microscope for imaging at cryogenic temperature. In one case, when it was observed that the sample did not readily stick to the grid surface, a glow discharged grid was used instead, solving the problem. In many cases, the grid was heated in a vacuum oven for a short period of time (15 min at 40°C) prior to plunging in liquid nitrogen in order to remove water and/or other volatile contaminants (Figure 3). Generally, only one grid was prepared per sample, and this was sufficient for data collection.

In a few instances, an alternative preparation method involving resuspending the sample in solvent, sonicating, and applying this crystal slurry to the grid was utilized. After drying, such grids were transferred to cryogenic temperatures. This method was employed when suitable crystals were not found using the dry application, but crystals were expected based on XRPD pre-screening results.

### Data Collection

Electron microscopy was performed using a Thermo Fisher Scientific (Hillsboro, OR, United States) Glacios Cryo Transmission Electron Microscope operated at 200 kV with the cryostage at −192°C. The microscope was equipped with a Thermo Fisher Scientific CETA-D detector. The camera length was calibrated using gold-palladium diffraction patterns collected using Grating Replica grids with Crossed Lines (EMS catalog #80051). Diffraction datasets were collected under parallel illumination conditions with a very low dose of ∼0.1 e^–^/Å^2^/s. A 20 μm condenser aperture was used during data collection, resulting in a ∼0.6 μm diameter beam on the sample. High gun lens (7.1) and spot size (10) settings were used to reduce the dose rate. The Leginon software package, adapted to collect MicroED data as described previously (Cheng et al., 2021), was used for all data collection. Briefly, crystal selection based on low magnification imaging mode images was carried out manually by the operator. Leginon provides the option to briefly test the diffraction of selected targets while queuing up targets for automated data collection. Automated data collection involves moving to and centering each target in imaging mode followed by recording the diffraction tilt series through the TEM User Interface with the camera set to record continuously in rolling shutter mode with 2 × 2 binning. This acquisition was synchronized with a continuous stage tilting function.

Diffraction datasets were then uploaded into the Leginon database with an approximate tilt angle assignment and could be viewed from anywhere in real time using the web-based Image Viewer. The MRC format files (^∗^.mrc) were converted to SMV format files (^∗^.img) with appropriate header information needed for data processing. An offset was applied to remove negative pixel values and is reported in the image header as LEGINON_OFFSET. A suggested pedestal value (IMAGE_PEDESTAL) is given in the image header and corresponds to the largest minimum pixel values across the dataset after applying the offset. A DIALS specific format class can then be used to optimally handle the images^1^.

### Data Processing

All datasets were initially processed with DIALS using default settings appropriate for electron diffraction data in a semi-automated fashion (Clabbers et al., 2018; Winter et al., 2018). The best datasets were selected based on various quality indicators (unit cell refinement, CC_1/2_, *R*_*meas*_, *I*/*σI*, etc.) for manual reprocessing. During manual processing, frames lacking reflections or exhibiting obvious radiation damage were removed from each dataset. These manually processed datasets were then fed into dials.cosym and scaled together in DIALS (Clabbers et al., 2018; Winter et al., 2018). Various combinations of datasets were evaluated with the goal of maximizing completeness and maintaining high redundancy while keeping favorable merging statistics. In most cases, these scaled, unmerged intensities were then trimmed to 0.9 Å regardless of merging statistics and then examined in XPREP (Sheldrick, 2008) before passing into SHELXT (Sheldrick, 2015b) and/or SHELXD (Schneider and Sheldrick, 2002) for phasing. Challenging cases typically required SHELXD with a high number of global tries. In many cases, it was necessary to attempt phasing from different combinations of datasets or in a number of possible space groups. When phasing was not possible in SHELXD, molecular replacement (MR) was employed. For MR, data were merged in AIMLESS (Evans, 2006; Winn et al., 2011) and then PHASER (McCoy et al., 2007) was employed with manually generated search models.

Refinement was carried out with SHELXL (Sheldrick, 2008) in Olex2 (Dolomanov et al., 2009) or REFMAC5 (Murshudov et al., 2011) and Coot (Emsley et al., 2010), depending on the phasing method. For structures refined with SHELXL, electron scattering factors were employed and riding hydrogens were added with the interatomic distances used for neutron scattering data (Gruene et al., 2018). The final resolution of the structure was established based on *R* factors and Goodness of Fit (GooF), and higher resolution reflections were excluded at this stage of data processing when appropriate. For all structures and values reported in this paper, the extinction parameter was not refined, which may be contributing to the higher *R*-factors relative to X-ray derived structures at the same resolution.

### Progesterone

**FIGURE 4:**
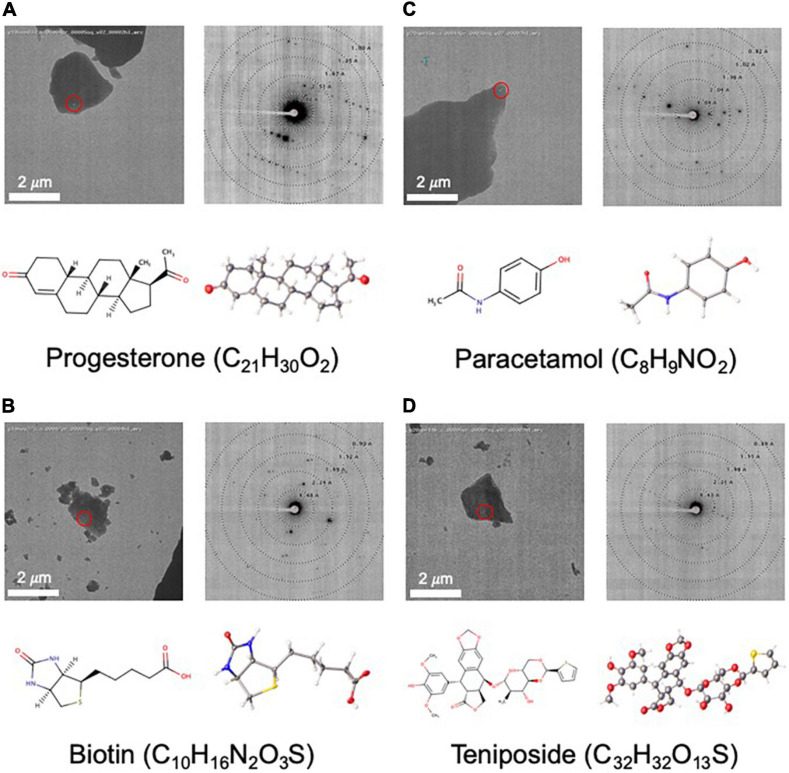
Structures determined by NIS as internal controls **(A–C)** and as a proof of concept for a more challenging sample **(D)**. For each panel, an image of a targeted crystal is shown with the beam diameter shown as a red circle, along with an exemplary diffraction image with overlaid resolution rings. Below this, the 2D structure is shown alongside the MicroED structure with atoms displayed as thermal ellipsoids. Note that no distinction is made between single or double bonds in the thermal ellipsoid image. The common name and chemical formula are also given.

Progesterone (Figure 4A) was obtained from Sigma-Aldrich (MDL Number: MFCD00003658). One grid was prepared using the dry application method as described above. Four diffraction datasets were collected with a camera length of 1,186.5 mm. All datasets were processed and combined as described above and phasing was carried out in SHELXT. Table 1 reports the final statistics. Raw data is available on Zenodo (10.5281/zenodo.3905397) and the final structure can be accessed from the CCDC (2084744).

**TABLE 1:** Summary of the data collection, reduction, and refinement statistics for four structures determined by NIS.

	Progesterone	Biotin	Paracetamol	Teniposide
	C_21_H_30_O_2_	C_10_H_16_N_2_O_3_S	C_8_H_9_NO_2_	C_32_H_32_O_13_S
**Data collection**				
Oscillation per frame (^*o*^)	0.9	0.89	0.89	0.89
Camera length (mm)	1,186.5	1,065.7	955.8	1,065.7
Datasets combined	4	4	4	6
Temperature (^*o*^C)	-192	-192	-192	-192
Accelerating voltage (kV)	200	200	200	200
Wavelength (Å)	0.02501	0.02501	0.02501	0.02501
Dose (e^–^/Å^2^/^*o*^)	∼0.045	∼0.022	∼0.022	∼0.022
**Data reduction**				
Space group	*P*2_1_2_1_2_1_	*P*2_1_2_1_2_1_	*P*2_1_/*n*	*P*2_1_2_1_2_1_
Unit cell a, b, c (Å)	10.28, 12.55, 13.50	5.14, 10.36, 20.80	7.07, 9.19, 11.49	9.27, 15.42, 20.50
Unit cell α, β, γ (^*o*^)	90, 90, 90	90, 90, 90	90, 98.64, 90	90, 90, 90
Resolution (Å)^*a*^	13.60–1.05 (1.07–1.05)	20.83–0.85 (0.87–0.85)	11.42–0.80 (0.81–0.80)	20.49–1.20 (1.22–1.20)
Total reflections^*a*^	10,900 (421)	11,275 (231)	10,031 (331)	15,834 (792)
Unique reflections^*a*^	949 (43)	1,125 (47)	1,350 (67)	1,074 (51)
Completeness (%)^*a*^	100.0 (100.0)	97.9 (82.5)	84.0 (82.7)	100.0 (100.0)
Multiplicity	11.5 (9.8)	10.0 (4.9)	7.4 (4.9)	14.7 (15.5)
Mean *I*/*σI*^*a*^	8.5 (1.2)	5.4 (0.2)	3.8 (0.4)	6.7 (1.0)
R_*meas*_^*a*^	0.25 (1.25)	0.26 (1.62)	0.30 (1.00)	0.26 (1.35)
R_*pim*_^*a*^	0.07 (0.38)	0.08 (0.47)	0.10 (0.43)	0.07 (0.33)
CC_1/2_^*a*^	0.993 (0.675)	0.988 (0.471)	0.975 (0.527)	0.995 (0.733)
**Refinement**				
Z/Z′	4/1	4/1	4/1	4/1
Total Reflections	10,209	10,626	9,049	14,062
*R*_*int*_/*R*_*sigma*_	0.22/0.16	0.23/0.38	0.26/0.32	0.25/0.20
Data/restrains/parameters	1,572/156/211	1,825/102/146	1,240/141/102	1,776/329/396
*R*_1_/*wR*_2_ (all data)	0.16/0.29	0.22/0.37	0.29/0.46	0.17/0.28
*R*_1_/*wR*_2_ [*I* ≥ 2σ(*I*)]	0.12/0.27	0.14/0.35	0.22/0.44	0.11/0.26
GooF	0.991	1.030	1.165	0.973

*^a^Numbers in parentheses refer to the highest resolution shell.*

### Biotin

Biotin (Figure 4B) was obtained from TCI (MDL Number: MFCD00005541). One grid was prepared using the dry application method as described above. Twelve datasets were collected with a camera length of 1,065.7 mm and data were processed as described above. Data from the best four crystals were combined and phasing was carried out in SHELXT. Table 1 reports the final statistics. Raw data is available on Zenodo (10.3389/fmolb.2021.648603) and the final structure can be accessed from the CCDC (2083844).

### Paracetamol

Paracetamol (Figure 4C) was extracted from Tylenol^®^ using hot acetone and filtration. Once the crystals were dry, one grid was prepared using the dry application method as described above. Forty-nine particles/crystals were assessed for diffraction quality using Leginon Preview Mode. Seventeen datasets were collected with a camera length of 955.8 mm and data were processed as described above. Eleven datasets failed at the indexing step and were discarded. Of the remaining six datasets, only four had the expected unit cell. These four datasets were combined and phased with SHELXT. Table 1 reports the final statistics. Raw data is available on Zenodo (10.5281/zenodo.4737931) and the final structure can be accessed from the CCDC (2084732).

### Teniposide

Teniposide (Figure 4D) was obtained from Enzo (CAS Number: 29767-20-2). One grid was prepared using the dry application method as described above. Eighty-eight crystals were assessed with Leginon Preview Mode and from these forty-four datasets were collected. Data were processed as described above. Ten datasets failed at the indexing stage and were discarded. Three indexed in a different crystal system and were also discarded. Of the remaining thirty-one data sets, the six best were combined. These data could not be phased by SHELXT and instead were solved by SHELXD (Sheldrick, 2008; Usón and Sheldrick, 2018) using all data to 0.9 Å. At the refinement stage, the final resolution was trimmed to 1.2 Å. Table 1 reports the final statistics. Raw data is available on Zenodo (10.5281/zenodo.3937739) and the final structure can be accessed from the CCDC (2015361).

### Pharmaceutical Samples Submitted by Clients

Proprietary material was received from clients in powdered form. We requested that clients send at least 1 mg of material and that they pre-screen the sample for crystallinity using XRPD if possible. Many, but not all, samples were pre-screened by XRPD. Grids were prepared using the dry application method or using a liquid resuspension step, and occasionally a drying step was applied to remove water or other volatile contaminants. Diffraction screening and data collection was carried out for fifty-six samples with a camera length of 1,065.7 mm. Automated data reduction was carried out for all samples as described above to assess data quality and preliminary space group assignments were made using dials.cosym. Most clients preferred to process and phase their own data, but occasionally when a client lacked internal expertise or when a client had difficulty processing and phasing their data, NIS proceeded with phasing. Phasing was carried out for fourteen client samples by NIS. Seven of these structures were fully refined by NIS to publication quality, again to assist clients lacking internal expertise.

### Figure Images

For Figure 1B, electron diffraction structures from the PDB were found using their online search tools and manually curated. The CSD structures were found using a combination of the CSD Python API (Groom et al., 2016) and the online search tools, again paired with manual curation. The reciprocal space image in Figure 2 was generated using ViewHKL in CCP4 (Winn et al., 2011) and the density map was generated in Coot (Emsley et al., 2010). The 2D chemical diagrams in Figure 4 were generated in MarvinSketch (Marvin 20.4, ChemAxon^2^). Diffraction patterns with resolution rings in Figure 4 were generated with Adxv Version 1.9.14^3^. ORTEP diagrams in Figure 4 were generated using SHELXL in Olex2 (Sheldrick, 2008; Dolomanov et al., 2009).

## Results

### Pipeline Validation (Structures of Progesterone, Biotin, and Paracetamol)

As part of our pipeline validation process, we determined the structures of three compounds, progesterone, biotin, and paracetamol (Figures 4A–C), that had already been solved by single crystal X-ray crystallography (Naumov et al., 1998; Shikii et al., 2004; Altaf and Stoeckli-Evans, 2013) and MicroED (Gruene et al., 2018; Jones et al., 2018). In each of these cases, data collection took less than 2 h, and structure solution was possible in less than 1 day using only SHELXT. Compared to the client submitted small molecule structures solved at NIS, these validation targets were very easy to solve. Additionally, these structures showed highly encouraging statistics for MicroED structures (low *R*-factor and GooF values close to 1).

### Teniposide Structure

As a proof of concept for a more challenging target, we used MicroED to determine the novel structure of teniposide (Figure 4D), an anti-cancer drug that has been on the US market for almost two decades. Crystal diffraction quality varied greatly from crystal to crystal, so Legion Diffraction Preview Mode was very useful for identifying higher quality crystals. Manual targeting and diffraction screening of almost one hundred crystals took about 3 h and automated data collection of approximately fifty datasets took about 1.5 h. Manual data processing for this sample proved to be fairly challenging. These crystals exhibited radiation damage, making it necessary to trim out affected frames and necessitating combining data from multiple crystals (six). Structure solution using SHELXT was unsuccessful and ultimately SHELXD was needed to successfully phase this data. While, structure solution was possible in 1 day, the data processing was not trivial and required a highly skilled crystallographer. The final structure has *R*_1_/w*R*_2_ values for all data of 0.17/0.28 [0.11/0.26 for data with I ≥ 2σ(I)] and a GooF of 0.973.

### Data Collection for Pharmaceutical Samples

**FIGURE 5:**
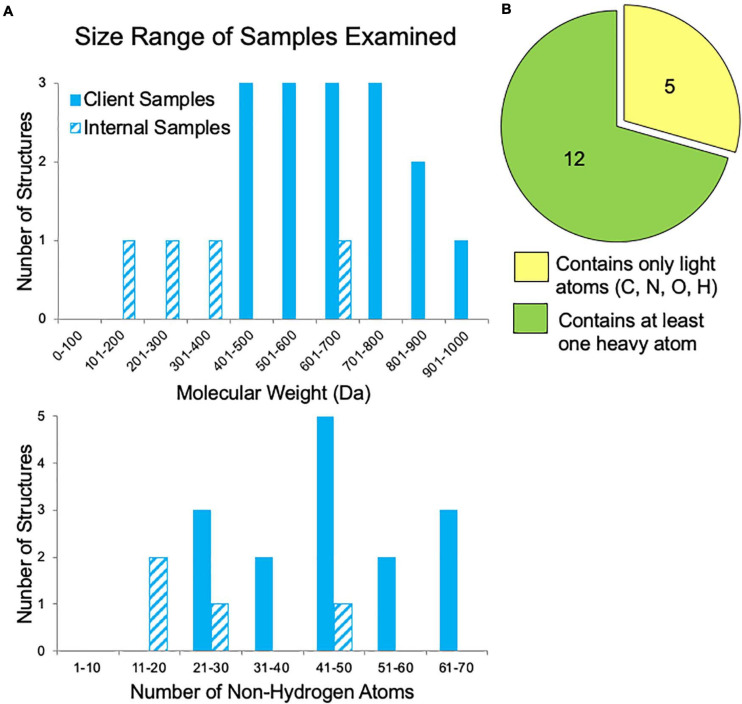
Sample characteristics for internal samples (progesterone, biotin, paracetamol, and teniposide) and a subset of client samples for which these data were known. **(A)** The range of sample sizes examined in terms of molecular weight and the number of non-hydrogen atoms (e.g., teniposide, C_32_H_32_O_13_S, has 46 non-hydrogen atoms). **(B)** The distribution of samples containing only light atoms (carbon, nitrogen, oxygen, and/or hydrogen) and those with at least one heavy atom (anything with an element having an atomic number larger than oxygen, such as sulfur, phosphorous, or chlorine), which can make phasing by *ab initio* methods much easier.

In our first year of offering small molecule MicroED data collection and structure solution services to pharmaceutical clients, we examined fifty-six samples. The majority of these samples failed to grow sufficiently large crystals for XRD studies despite attempts by experienced small molecule crystallographers. Additionally, for many samples, various NMR and MS experiments had already been performed, yet questions regarding the 2D and/or 3D structure still remained. Samples submitted by clients for which the molecular weight was disclosed ranged in size from 400 to 1,000 Da, corresponding to 20–70 non-hydrogen atoms (Figure 5A). These are fairly large “small molecules”, which possibly contributed to their inability to be solved by traditional structural methods. Additionally, about one third of the samples contained only light atoms (carbon, nitrogen, oxygen, and hydrogen), which can make phasing by *ab initio* methods (SHELXT and SHELXD) more challenging if the resolution is poor or if there are other data quality issues (Figure 5B). Though anomalous signal is thought to be very minimal in electron diffraction, the presence of heavier atoms such as sulfur, chlorine, phosphorus, or bromine within a small molecule can be quite helpful for phasing. Heavy atom peaks, even without anomalous signal, are much easier to correctly locate by *ab initio* methods and can be used to bootstrap to a complete solution.

For samples considered “easy cases,” about 1 hour was spent finding crystals, screening them for diffraction quality and adding targets to the queue, and about 1 hour was spent automatically collecting 20 datasets (Figure 2). For more challenging cases, the grid might be removed for a heating step prior to re-insertion or the wet application grid preparation protocol might be followed after the dry application failed to generate suitable crystals (Figure 2). In cases presenting data processing challenges, long (four or more hours) or overnight data collections might be set up, collecting 50–150 datasets to ensure enough high-quality data was collected for structure solution (Figure 2).

### Aggregate Data Collection Results

**FIGURE 6:**
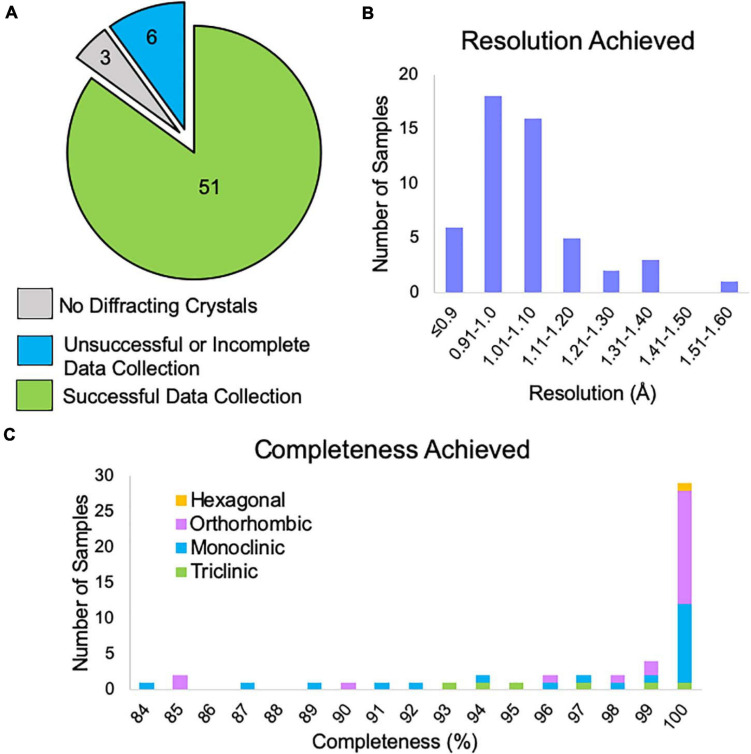
**(A)** The outcome of MicroED data collection at NIS for client and internal samples (progesterone, biotin, paracetamol, and teniposide). Three samples did not contain diffracting crystals, but we note that XRPD pre-screening was not performed for these samples prior to submission. Six samples did not yield enough data suitable for structure solution and likely need additional microscope time, possibly with an alternative grid preparation method. Fifty-one samples produced data that should be sufficient for structure solution. **(B)** For the 51 successful data collections, the approximate resolution limits are shown. This resolution estimate is based on automated processing results from DIALS for the highest resolution crystal. This value is based on CC_1/2_ ≥ 33%. We note that the final resolution after processing by a trained crystallographer may differ from this cutoff. **(C)** Of these data collections, the overall completeness based on combining all crystals is shown broken down by Bravais lattice type. Automated processing in DIALS was carried out in the space group suggested by dials.cosym with all crystals for which integration was successful. Overall completeness is shown to the resolution limit determined in panel **(B)**. The single hexagonal sample was in space group P6_4_.

Of the samples examined by NIS (both internal and client samples), 85% produced data expected to be suitable for structure solution (Figure 6A). The remaining samples either showed no diffraction or had data collection curtailed due to microscope time constraints. There were three samples that did not appear to contain crystals and after consultation with the client we found that XRPD was not collected from these samples prior to submission, leading us to suspect that these samples were not in fact crystalline. We note that, all samples for which XRPD was used as a pre-screening tool contained diffracting crystals. One of the six “unsuccessful” samples had obvious water contamination issues (frozen puddles of water partially dissolving the crystals). We have previously rescued such samples by heating the sample in a vacuum oven for a brief period of time prior to plunging into liquid nitrogen, and it is likely that this sample could also be rescued in this way. Another of the “unsuccessful” samples did not contain any diffracting crystals following our typical dry application to the TEM grid. We subsequently found that resuspension of this sample in a solvent, applying the suspension to the TEM grid and allowing it to dry resulted in diffracting crystals. The remaining four samples contained diffracting crystals with a reasonable unit cell large enough to accommodate the compound of interest, but collection of a sufficiently large number of datasets was not possible during the client’s allotted microscope time. Many of our clients book single microscope days in which we try to collect data from as many samples as possible, which can lead to incomplete data collection for the last sample in the queue. Of course, these remaining samples can easily be revisited during the next scheduled microscope time.

Data reduction for all samples that produced viable data revealed that 78% of samples achieved resolution better than 1.1 Å based on a metric of CC_1/2_ ≥ 33% (Figure 6B).

After combining data from multiple crystals, 78% of samples reached completeness >95%, while 57% of samples achieved 100% completeness (Figure 6C). We note that low completeness did not hinder structure solution for any of the samples phased at NIS. For lower completeness samples, we suspect that this is due to preferred orientation of the crystal on the grid and if so, it is doubtful that additional data collection from these grids would produce more complete data.

### Aggregate Phasing and Refinement Results

**FIGURE 7:**
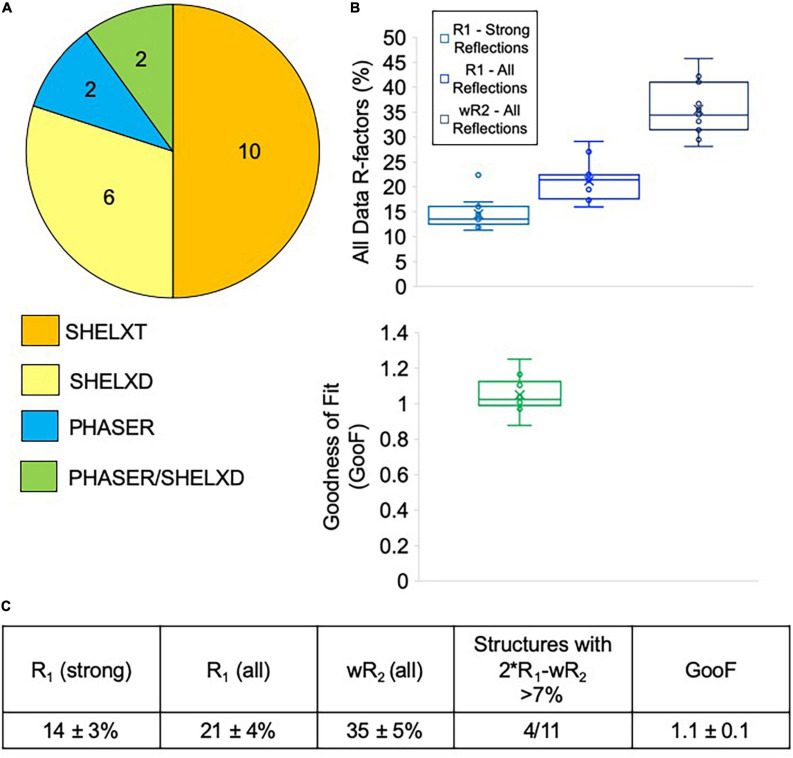
**(A)** Phasing method used for structures solved at NIS (internal and client samples). Generally, SHELXT was attempted first, followed by SHELXD, followed by PHASER. For two samples, a combination of SHELXD and PHASER were used. **(B)** Average refinement statistics for structures determined and refined at NIS shown as boxplots. Note that only structures solved with SHELXT and SHELXD are included in this analysis. **(C)** Average refinement statistics for structures determined and refined at NIS.

Most of the data collected at NIS was processed externally by our clients, and we do not have data on how many of these datasets were successfully phased and refined. We did, however, process data from sixteen client samples, all of which we were able to successfully phase. Data processing difficulties varied widely for client samples, with some taking an hour or two to solve, while others required several rounds of iterative effort over multiple days (Figure 7A). Of the twenty internal and client samples determined by NIS, ten samples were considered easy to process and could be readily solved with SHELXT. The remaining ten samples had to be solved with SHELXD (six samples) or PHASER (two samples), or a combination of both PHASER and SHELXD (two samples). Structures requiring MR in PHASER generally were the result of low resolution (worse than 1.1 Å) and/or a poor data-to-parameter ratio (combination of low completeness and high Z’–number of molecules in the asymmetric unit). For the more challenging cases, we found that it helped to include data to 0.9 Å, regardless of the actual resolution cutoff suggested by CC_1/2_, and increase the cycles in SHELXT/D. Additionally, reducing the symmetry to P1 and phasing with SHELXD was extremely helpful for samples suffering from pseudo translational symmetry issues that might or might not be apparent during data processing. Once the structure was solved in P1, the actual crystal symmetry could be deduced by examining the model, and the data could then be re-solved in the correct space group. For PHASER, we found that success generally relied on having a very accurate search model (extremely low RMSD with the final refined structure) that represented the majority of the compound by mass.

Seven of the client structures determined by NIS were also fully refined by NIS at the request of clients lacking this expertise. Taken together with the four structures detailed in Table 1, structures collected and refined by NIS appear to achieve very encouraging refinement statistics when compared with other published MicroED structures. NIS structures achieved an average *R*_1_/*wR*_2_ for all data of 14 ± 3%/35 ± 5% and an average GooF value of 1.1 ± 0.1 (Figure 7B). Ideally, *wR*_2_ should be about the same magnitude as two times *R*_1_ and deviations from this can indicate problems with the data or with the data handling. For the samples included in this analysis, only four out of eleven produced larger than 7% difference between 2^∗^*R*_1_-*wR*_2_ (Figure 7C). Overall, we are very encouraged by these refinement statistics given the resolution of our data and the current challenges posed by processing electron diffraction data.

## Discussion

Microcrystal electron diffraction is becoming an attractive alternative structure determination technique for proteins (Shi et al., 2016) and small molecules (Gruene et al., 2018; Jones et al., 2018). Since electron diffraction allows for data collection from crystals orders of magnitude smaller than those required for single-crystal XRD, this technique can deliver structures of challenging targets for which large crystals are not available. This is especially exciting for small molecules, many of which readily form microcrystals, especially during the purification process. In such cases, MicroED often eliminates the need for crystallization screening, greatly reducing the sample requirements. Taken together, MicroED is poised to assist in the drug development pipeline at every step along the way: from the discovery phase, where sample quantities are generally too limited for crystallization trials, to process chemistry where structures of reaction products and by-products can guide synthesis strategies, and to formulation chemistry where the crystallization space of the API can be explored, better understood and engineered.

In medicinal and process chemistry, MicroED is poised to transform workflows making accurate structure determination a routine and essential tool, rather than an exotic, “nice-to-have” tool. Nevertheless, MicroED adoption has been slow, especially in industrial settings, where widespread adoption has been inhibited by steep requirements for instrumentation, infrastructure and expertise, especially when the number of projects may not justify the initial investment. Improvements in access to facilities, in speed, reliability and automation for data collection and processing, and in structure solution and refinement, are needed for MicroED to fulfill its potential of becoming a routine tool in small molecule characterization. We have built and demonstrated an efficient, semi-automated MicroED pipeline that can be used to image multiple samples per day and solve their structures on a timeline of 1 day to several days, depending on the complexity of the sample data.

### Progesterone, Biotin, and Paracetamol Structures

To validate our MicroED setup, we determined the structures of three compounds that had already been solved by single crystal XRD and MicroED: progesterone, biotin and paracetamol (Figures 4A–C). Data for all three compounds achieved similar resolution and data reduction statistics compared to previous MicroED efforts (Gruene et al., 2018; Jones et al., 2018). The structures of all three compounds were solved with SHELXT (Sheldrick, 2008, 2015b) and refined with SHELXL (Sheldrick, 2015a) as implemented in Olex2 (Dolomanov et al., 2009), similarly to what was already reported. Statistics for the refined structures are fairly comparable to previously reported MicroED structures, with one notable difference being the value of the GooF parameter which in all the NIS structures is closer to the ideal value of 1 (Figure 7C and Table 1), whereas previous groups have obtained values closer to 2 (Gruene et al., 2018; Jones et al., 2018).

### Teniposide Structure

Teniposide [epipodophyllotoxin, 4′- demethyl-, 9-(4,6-*O*-2-thenylidene-beta-D-glucopyranoside)] is a semi-synthetic derivative of podophyllotoxin. This organic heterotetracyclic compound is found in the roots and rhizomes of Podophyllum species. It has been shown to exhibit antitumor activity via inhibition of DNA synthesis, achieved by forming a complex with topoisomerase II and DNA (Bh, 1992). This complex induces breaks in double-stranded DNA and prevents repair by topoisomerase II. Accumulated breaks in DNA prevent cells from entering into the mitotic phase of the cell cycle, and lead to cell death. Teniposide acts primarily in the G2 and S phases of the cell cycle. In the United States, teniposide is currently used in the treatment of children with Acute Lymphocytic Leukemia (ALL) that has not improved or that has worsened after treatment with other medications (Dahl et al., 1985).

Despite being on the market for almost two decades, there is no single-crystal X-ray structure of the solid form of teniposide deposited in the CCDC. It is possible that such a structure has been determined, but not deposited, by industrial researchers. We speculate that the lack of such a structure may be due to difficulties growing sufficiently large crystals for X-ray studies. For this reason, teniposide was chosen as a proof of concept target for our MicroED pipeline. Given the high cost and technical challenges associated with MicroED, we wanted to demonstrate that this technique could solve structures not easily accessible to XRD. Teniposide was also an attractive target given its size and complexity, especially considering that most of the early small molecule MicroED structures published in the literature had molecular weights of less than 400 Da, which generally makes structure solution easier. In contrast, all of the samples submitted by our industrial clients were of compounds with molecular weights more than 400 Da (Figure 5A). Thus, it was important for us to demonstrate that MicroED is a viable option for these larger compounds. While data collection and data processing were both more challenging for teniposide compared to progesterone, biotin, and paracetamol, we have shown that structure solution was still possible in a single day.

**FIGURE 8:**
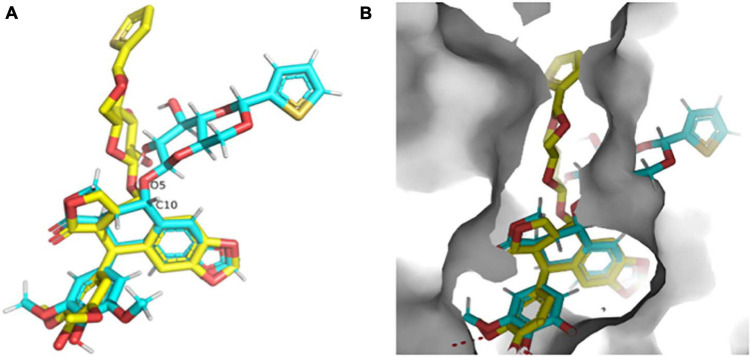
**(A)** Overlay of the teniposide MicroED structure determined in this paper (cyan carbon atoms, CCDC KUXJUL) and the structure of teniposide bound to human serum albumin (HSA) from PDB 4L9Q (yellow carbon atoms). **(B)** Overlay of the two teniposide molecules (same coloring scheme as in panel **(A)** within the HSA binding pocket demonstrating that the conformation observed in solid state form is distinct from the conformation required for protein binding. UCSF Chimera was used to generate these images (Pettersen et al., 2004).

The high-resolution structure of the solid form of teniposide solved by MicroED is chemically consistent with a structure of teniposide bound to human serum albumin (HSA) (PDB entry 4L9Q; Figure 8A). The biggest difference is in the torsional angle at the C10-O5 bond (−134° in 4L9Q versus −69° in the structure reported here, Figure 8A). This bond is expected to be able to freely rotate and the change in torsional angle is likely the result of the constraints induced by the surrounding protein atoms in the HSA-bound form versus the constraints imposed in the solid form of the compound (Figure 8B).

### Aggregate Data Collection and Processing Outcomes

At NIS, we were able to collect data suitable for structure solution for the majority of samples submitted (Figure 6A) and for data we have been asked to process we have had a 100% success rate for structure solution. This is very encouraging given the large size of many of these samples (Figure 5A) and the lack of heavy atoms (sulfur, chlorine, etc.) in 30% of the samples examined (Figure 5B), which is thought to make phasing by *ab initio* methods more challenging. Additionally, nearly 80% of samples collected at NIS achieved at least 1.1 Å resolution (Figure 6B). This is important given that 1.1 Å is generally considered the point beyond which phasing by direct and dual space methods becomes exceedingly difficult (Sheldrick et al., 1993). Low completeness in many MicroED datasets is often cited as a concern for this method. We found that our pipeline produced at least 84% completeness for all samples for which data was collected, with more than half achieving 100% completeness (Figure 6C). We also note that low completeness (as low as 84%) did not hinder structure solution or map interpretation for data processed internally. The data for paracetamol were only 84% complete overall, yet the map for this compound was reasonably isotropic and easily interpreted. We were also encouraged by our average refinement statistics, which compare very favorably with MicroED structures in the literature (Figures 7B,C). Given these results we are satisfied that our MicroED pipeline is quite robust. We anticipate that for any small molecule sample proven to be crystalline by XRPD data, we should be able to solve a single crystal MicroED structure using 2–4 h of microscope time and one to several days of data processing time.

### Leveraging the Relationship Between MicroED and XRPD

**FIGURE 9:**
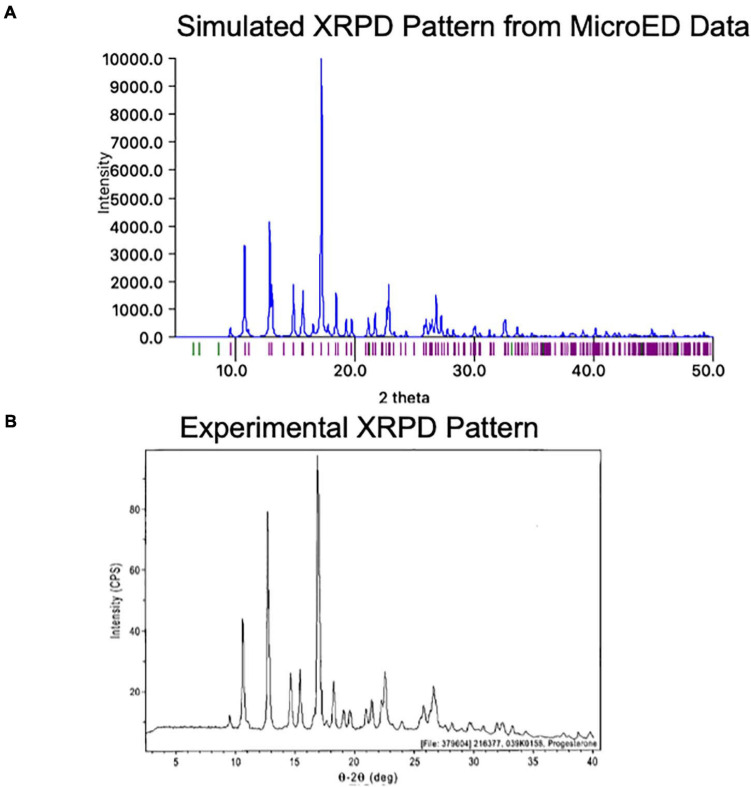
**(A)** The MicroED structure of progesterone was used to generate a theoretical X-ray powder diffraction (XRPD) pattern at a wavelength of 1.54 Å using Mercury (Macrae et al., 2020). Intensity values are relative to the highest peak. **(B)** The experimentally measured X-ray powder diffraction (XRPD) pattern for Form I of progesterone is shown roughly lined up to the pattern in panel **(A)**^4^. We note that some differences may be due to the different temperatures at which each pattern was measured (the MicroED pattern was collected at −192°C while the experimental pattern was likely collected at room temperature).

One exciting feature of MicroED data is that the final coordinates produced are practically indistinguishable from those produced by X-ray methods. Because of this, a structure generated by MicroED can be readily used to generate a predicted XPRD pattern for its crystal form at a given X-ray wavelength, effectively generating an XRPD fingerprint. This fingerprint can be very useful as it can be compared to experimentally measured XRPD data (Figure 9). Comparing an experimentally measured XRPD spectra to the known MicroED-based spectra can quickly confirm the identity of a reaction product, such as might be produced during synthesis optimization. Additionally, XRPD from a mixture of crystals can be resolved into the relative ratios of these crystal forms in the mixture, as long as the expected XRPD fingerprints for each compound is known. In this way, XRPD screening based on MicroED data for a low abundance contaminant can be very helpful in optimizing synthesis and purification strategies.

### Practical Notes for Establishing a MicroED Pipeline

There are many individuals interested in establishing their own MicroED pipeline, especially one centered around existing microscope equipment. While evaluating and comparing hardware requirements for MicroED is outside the scope of this paper, we do have some practical advice for newcomers to the field. It is important to keep in mind that compounds of interest to the pharmaceutical industry that fail to be solved by XRD often pose significant challenges for MicroED data processing.

While examples of structures solved using lower end microscopes and cameras exist in the literature, we anticipate that many large organic crystals inaccessible by XRD might be impossible to solve with lower quality MicroED data. Data collected at NIS using the CETA camera prior to upgrading to the CETA-D camera was of lower quality, and while we were able to use it to solve the structure of paracetamol, we are doubtful that data from such a camera would have been sufficient to solve a more challenging crystal structure like teniposide using *ab initio* methods. We suggest that those interested in setting up their own MicroED facility first collect data from easy to solve crystals such as paracetamol or biotin but then try to solve a more difficult case, such as teniposide, to get a sense of whether or not their equipment and data processing pipeline can in practice deliver structures that require MicroED techniques. At this point in time, MicroED is relatively expensive and often poses significant data processing challenges. Until these two issues are addressed, we suggest that MicroED be reserved for samples that absolutely require this technique because they fail to produce large crystals or because the sample quantity is limiting.

### Future of the Field

Our experience collecting data from over fifty pharmaceutical small molecule samples provides high optimism about the role MicroED will play in the future for drug discovery and development. We have demonstrated that structure determination by a MicroED service provider can be viable way for industrial researchers to gain access to this cutting-edge technology and leverage it to expedite research endeavors. We hope that future efforts in the field will yield better data processing tools for electron diffraction data, making it easier for newcomers to the field to process their own data which should in turn expedite results and reduce costs.

## Data Availability Statement

All raw datasets contributing to the structures of progesterone, biotin, paracetamol, and teniposide have been deposited to the Zenodo, the open access public data repository as part of the 3DED/MicroED datasets community. The final structures (coordinates, reflections, and structure factors) of progesterone, biotin, paracetamol, and teniposide have been deposited in the Cambridge Crystallographic Data Center (CCDC), a part of the Cambridge Structural Database (CSD), a public data repository. Zenodo DOI and CCDC numbers are listed in the text.

## Author Contributions

JB, GS, and BC conceptualized and wrote the manuscript. AC, JB, and TN established data collection strategies. JB, KL, MMy, BR, TN, NC, NP, PM, TW, MMs, and CH prepared the samples and collected the data. JB, GS, BM, and BR processed the data. AC, TG, SD, BR, JB, DW, and ST automated data handling, processing and analysis. BW provided some samples, including the sample shown in Figure 3. CP helped secure the funding. All authors contributed to the article and approved the submitted version.

## Conflict of Interest

JB, GS, AC, TG, SD, BR, TN, KL, MMy, NC, NP, PM, TW, MMs, CH, ST, CP, and BC are or have been employed by NanoImaging Services, a commercial supplier of electron microscopy services to the biopharmaceutical and biotechnology industries. BW is employed by the company Biogen. The remaining authors declare that the research was conducted in the absence of any commercial or financial relationships that could be construed as a potential conflict of interest.

## Footnotes

1 https://raw.githubusercontent.com/dials/dxtbx_ED_formats/master/FormatSMVCetaD_TUI.py

2 https://www.chemaxon.com

3 Copyright© 1994–2019 Andrew Arvai, The Scripps Research Institute.

4 https://patentimages.storage.googleapis.com/b3/6d/09/db2cfde1e296ab/US20170101433A1.pdf
